# Finite element analysis of anterior spanning attachment devices for supporting biomechanical stability in diaphyseal femoral periprosthetic fracture fixation

**DOI:** 10.1038/s41598-025-11174-9

**Published:** 2025-07-25

**Authors:** Markus Heinecke, Stefan Schwan, Immanuel Ries, Ram Kumar Jayakumar, Filippo Migliorini, Thomas Mendel

**Affiliations:** 1 Chair of Orthopedics of Jena University Hospital, Campus Eisenberg, German Center for Orthopedics, Jena University Hospital, Klosterlausnitzer Strasse 81, 07607 Eisenberg, Germany; 2 Fraunhofer Institute for Microstructure of Materials and Systems IMWS, Walter-Hülse-Strasse 1, 06120 Halle (Saale), Germany; 3 Department of Engineering and Natural Sciences, Hochschule Merseburg, University of Applied Sciences, Eberhard-Leibnitz-Strasse 2, 06217 Merseburg, Germany; 4 Department of Orthopaedic and Trauma Surgery, Academic Hospital of Bolzano (SABES-ASDAA), Via Lorenz Böhler 5, 39100 Bolzano, Italy; 5 Department of Life Sciences, Health, and Health Professions, Link Campus University, Via del Casale Di San Pio V, 00165 Rome, Italy; 6 Department of Trauma and Reconstructive Surgery, BG Hospital Bergmannstrost Halle gGmbH, Merseburger Strasse 165, 06112 Halle (Saale), Germany; 7 Department of Trauma and Reconstructive Surgery, University Hospital of Halle, Martin-Luther University Halle-Wittenberg, Ernst-Grube-Street 40, 06097 Halle (Saale), Germany

**Keywords:** Finite element analyses, Anterior spanning attachment devices, Biomechanics, Periprosthetic fracture fixation, Medical research, Risk factors

## Abstract

The incidence of periprosthetic femoral fractures has increased in recent years. Osteosynthetic stabilisation is challenging, particularly for UCS IV.3-C fractures. Lateral plate osteosynthesis is the gold standard; however, it allows excessive vibration, leading to plate breakage. Orthogonal double plate osteosynthesis has been established but requires considerable intraoperative dissection of the anterior extensor muscle. This work aims to analyse newly developed plate designs that demonstrate adequate vibration behaviour, which, in turn, promotes callus healing and causes less soft tissue trauma than the plate constructs used to date. A hip prosthesis geometry and a parameterised volume geometry of a UCS IV.3-C type periprosthetic femur fracture were simulated to generate a finite element model. Additionally, three alternative design studies were developed to optimise an LCP®, and the various constructs were then investigated using a finite element model concerning comparative stress and deformation under static and dynamic loading and their influence on fracture gap expansion. Isolated lateral plate osteosynthesis (V1) and double plate osteosynthesis (V2) served as references. The alternative plate designs include a ventral frame at the fracture level (V3) or spanning the length of the lateral LCP® (V4). The fifth variant is a fulcrum support attached to the existing LCP® at the fracture level (V5). Compared with V1, V3 and V4 yielded comparable results, presenting greater stiffness and increased survival. The functionality of V5 shows nearly identical outcomes to those of V1. Here, failure with plastic deformation is already observed under static loading, which does not occur with V2 even under dynamic loading, thus representing the most stable construct, albeit one that does not permit adequate vibration behaviour. For V3 and V4, optimal strain behaviour in the fracture gap is also evident after load application. Alternative implant design variants with an additional anterior frame lead to reduced deformation and failure of fixation in UCS IV.3-C periprosthetic femur fractures. In addition to double plate osteosynthesis, alternative plate constructs exhibit optimal strain behaviour conducive to callus fracture healing. Furthermore, the selected designs decrease the required dissection of the quadriceps muscle.

## Introduction

The incidence of periprosthetic femur fractures has risen in recent years, owing to the increased number of hip endoprostheses implanted and the desire of ageing adults to remain active despite declines in bone quality^1–3^. The reported rate of periprosthetic femoral fractures after cementless hip arthroplasty ranges from 0.4 to 5.4%^4^. For revision arthroplasties, this rate is as high as 20.9%^4^.

The primary aim of prompt treatment for a periprosthetic femoral fracture is to ensure adequate patient mobilisation and the ability to perform functional exercises as soon as possible without increasing the risk of secondary complications. Furthermore, fractures associated with a stable prosthesis should heal promptly. Osteosynthesis plays a crucial role in the healing process, particularly in UCS (unified classification system) type IV.C fractures, which are defined as having a stable prosthesis with the fracture located distal to the stem or cement mantle. Plate osteosynthetic fixation with preservation of the inserted prosthesis is the preferred method^5^. However, a notable challenge associated with the abovementioned method is the correct placement of the screws for secure anchoring of the plate in the proximal main fragment around the femoral stem. Various implants for periprosthetic femoral fractures have been developed to increase the mechanical stability and strength of the plate in the area where the prosthesis is placed^6–9^. Nevertheless, current studies on locking plates have reported a complication rate of up to 30%^10^. One major cause is the high impedance jump between the rigid, proximal main fragment in which the stem of the prosthesis is implanted and the distal main fragment with its usually reduced bone structure. For the fixation of a periprosthetic femoral fracture, it is, therefore, necessary to consider the anatomical characteristics and associated biomechanical properties of the femur.

The mechanical load axis of the femur induces tensile forces on the lateral side. In addition, owing to the antecurvature of the femur when standing or after lifting the leg or climbing stairs, bending forces, resulting in tensile loads, also act on the ventral side. The established method for osteosynthesis of a periprosthetic femoral fracture is lateral fixation, which allows the lateral tensile forces described above to be absorbed and converted. However, there are several issues associated with isolated lateral plate osteosynthesis. On the one hand, plate fixation failure is caused by cyclical loads; on the other hand, the mechanical properties of the plate can delay bone healing^11^. Furthermore, some currently available plates appear too weak to withstand the forces acting on them, resulting in breakage^12–14^. On the other hand, the lack of medial contact between the fragments in most cases leads to high vibration amplitudes at the fracture level, which can delay the healing of callous fractures, resulting in plate fixation failure^15^.

However, particularly for multi-fragmentary or medial fractures, fracture consolidation, such as secondary fracture healing, necessitates a certain degree of stretching for adequate callus formation. Numerous studies have indicated that an appropriate level of longitudinal movement of the femur leads to tissue elongation and stimulation of tissue differentiation, facilitating the healing of callous fractures through interfragmentary relative movement^16,17^. In contrast, rotational and shearing movements can delay fracture healing, potentially leading to pseudarthrosis^18^. The adverse effect of movement in these directions on longitudinal movement increases with the working length of the plate.

Plates attached anteriorly, in addition to the lateral plate, are intended to counteract the ventral bending forces, thereby reducing the vibration amplitude in the fracture area and making the construct stiffer. However, this technique necessitates extensive soft tissue dissection, where the extensor muscle must be detached from the anterior aspect of the femur. In corresponding biomechanical studies, fixation with this 90° plate is superior to isolated lateral plate osteosynthesis because the survival of the plate used in the former fixation method is prolonged compared to the latter, and the time to failure is extended^15,19,20^. However, the rigidity of this fixator compromises the periosteal blood supply, particularly in the fracture area. In the tension between the necessary mechanical stability and the desire to minimise harm to the surrounding soft tissue, an implant construct founded on a classic lateral locking compression plate (LCP®) should, therefore, effectively counteract the bending forces on the anterior side of the femur without requiring extensive soft tissue access. Hence, an "anterior tension band frame" for the LCP® was developed as part of this study. In this investigation, various plate designs were analysed using computer-aided design (CAD) and the finite element method (FEM), considering other material properties (stainless steel and titanium) in a model of type UCS IV.3-C periprosthetic femoral fractures, and the mechanical properties under static and dynamic loading are compared. The aim is to develop new plate designs and test them to determine which offers the best mechanical properties with adequate vibration behaviour and, consequently, a positive effect on callous healing according to Perren’s strain theory, as well as the longest service life in terms of cyclic load capacity when compared to current systems^21^. Ideally, this should minimise or eliminate the need for anterior soft tissue dissection on the femoral shaft.

## Methods

### Implantat designs

The implants were assessed using CT (BrightSpeed Performix 16 SI, General Electronic Healthcare Germany, Munich, Germany, slice thickness of 0.625 mm), and all imaging data were saved in "Digital Imaging and Communications in Medicine" (DICOM) format. The data were subsequently converted into Standard Triangulation Language (STL) format and finally imported into a CAD programme (CATIA Version 5, Dassault Systèmes, Vélizy-Villacoublay, France). From this, images of the implants and their adaptations were digitised. This process allowed for the creation of a total of five different fixation variants. The other constructs were then analysed using a finite element (FE) model to assess comparative stress and deformation under static and dynamic loading.

### Bone model with periprosthetic femur fracture

The Sawbone femur model (#3403 medium left, 4th generation, composite, Sawbone®, Vashon, Washington, USA) served as the bone model. The total hip endoprosthesis (CLS® Spotorno® Stem, ZimmerBiomet®) was positioned with an antetorsion of 14° in the proximal femur and inserted deeply enough that the head projection matched the anatomical centre of rotation of the hip joint. The mechanical-anatomical axis of the femur was assumed to be 6° physiological valgus and was aligned accordingly. The type C periprosthetic femoral fracture was located 10 mm below the end of the prosthesis stem in the femoral shaft and resulted in a wedge-shaped bone defect that was open 10 mm medially. The parameterised volume geometry of the bone and the fracture (type UCS IV.3-C) remained identical for all application variations.

### Finite element analysis and calculation principles

**Fig. 1:**
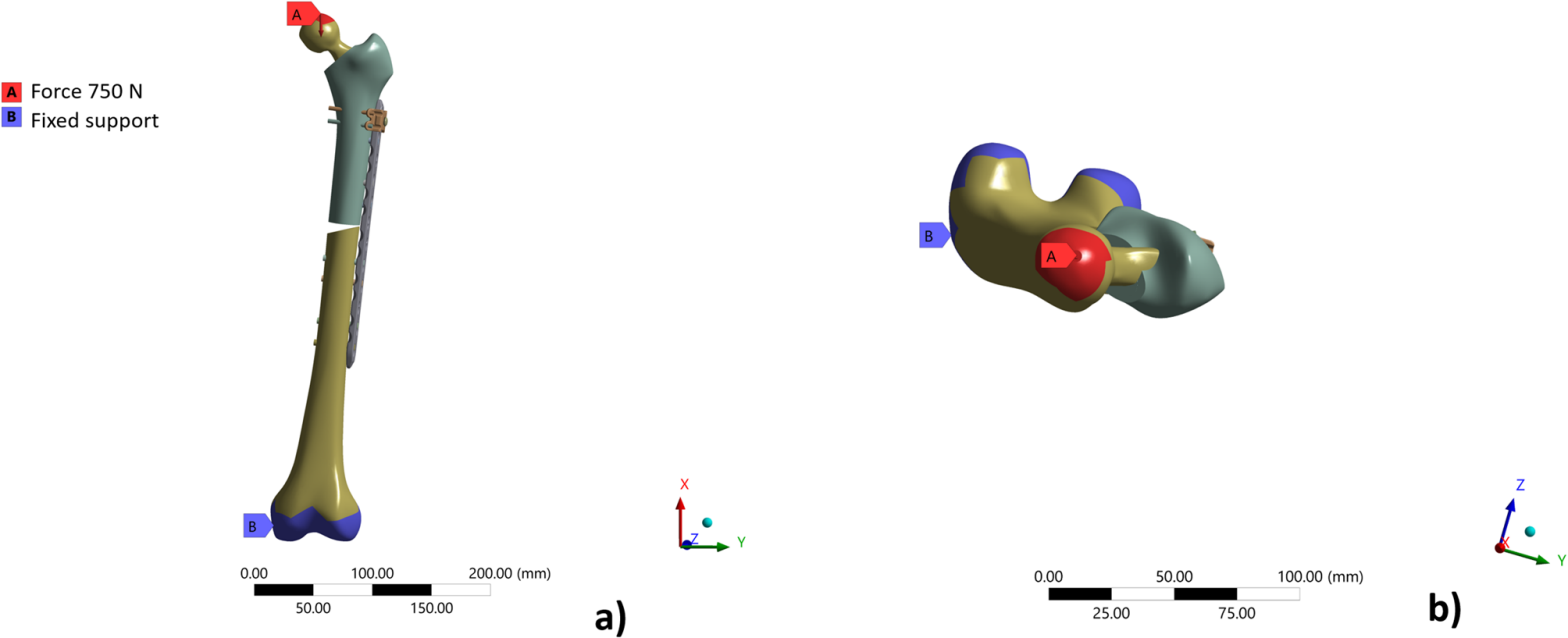
(**A**) Force of 750N; (**B**) fixed joint in coronal plane (**a**) and transversal plane (**b**).

The mechanical behaviour of five different fixation constructs was analysed using the finite element method. All systems were subjected to identical boundary conditions and loading scenarios. A fixed support was applied at the distal femoral condyles. Vertically in the x-direction, a force of 750 N (a body weight of 75 kg) was applied to a surface of the total endoprosthesis (TEP), which approximates the shape of a hemisphere and is intended to represent the contact area within the corresponding hip joint (Fig. 1).

**Fig. 2:**
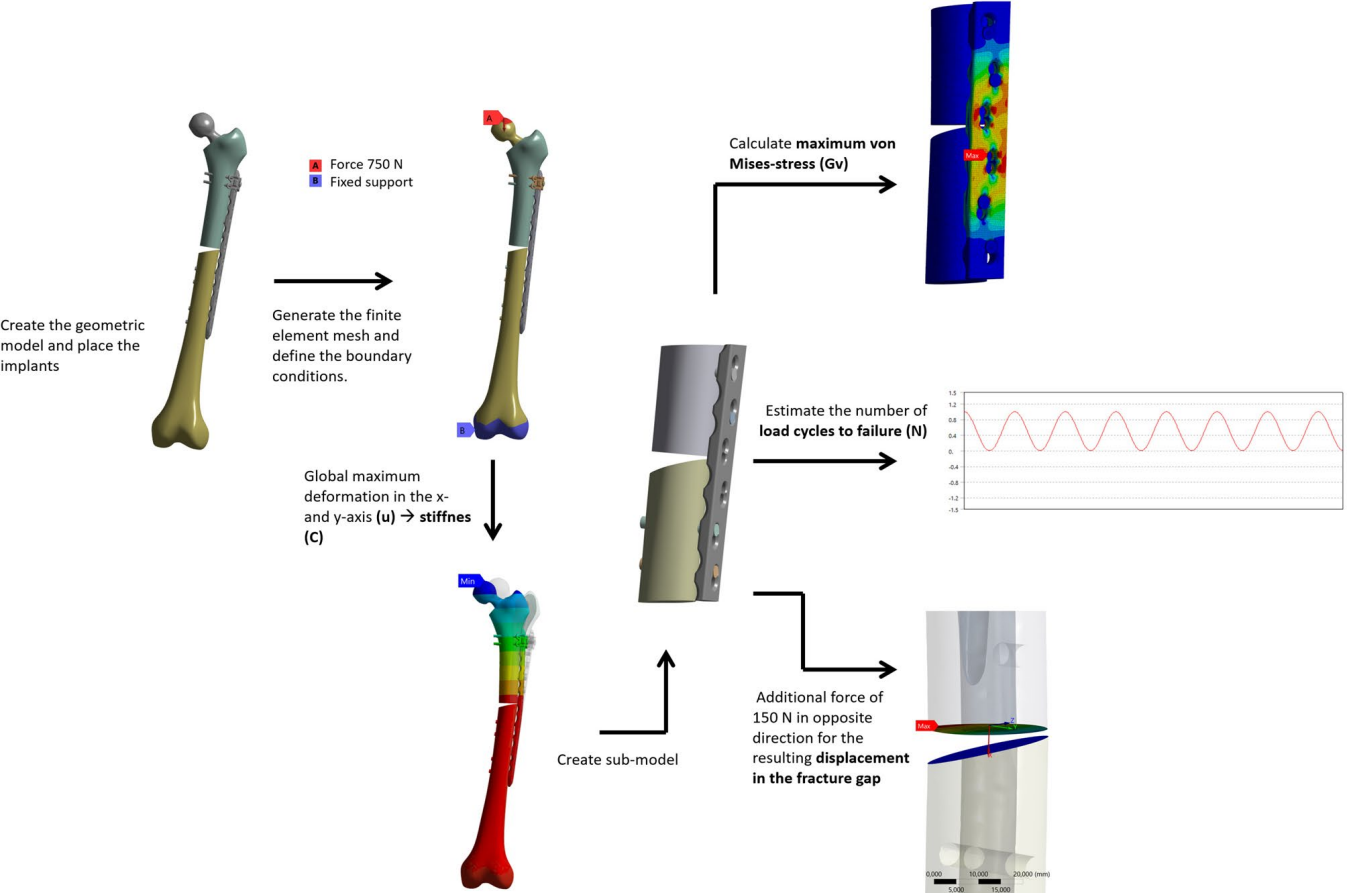
Different results of the FE-Analysis to compare the plate systems. Global Stiffness (C) from global deformation (u) at the top of the TEP, maximum von Mises Stress at the plate systems, the submodel and fracture-gap displacement.

To assess the mechanical properties, various results from the finite element analysis were evaluated, and configurations V1 to V5 were systematically compared. The results are distinguished between static and dynamic analyses (Fig. 2). The static analysis comprises the evaluation of global stiffness (C), calculated from the deformation (u) and the applied force, the maximum von Mises stress in the plate systems (Gv), and the displacement at the fracture gap. The dynamic analysis serves to estimate the fatigue life, expressed as the number of load cycles (N).

To determine the stiffness **(C)** of the complete system, the total displacement u_x_ and u_y_ in both the vertical x-direction (cranial-caudal) and horizontal y-direction (medial–lateral) are used and calculated as follows:

$${C}_{x,y}=\frac{F}{{u}_{x,y}}$$

The maximum deformation occurs at the top of the TEP.

For all results other than stiffness (C), a sub-model was applied. This allows for more accurate results due to a finer mesh in the region of interest (ROI), which corresponds to the plate systems located at the level of the fracture. The von Mises stress (Gv) was assessed based on sub-model analyses for each fixation variant. The maximum value observed within each sub-model was taken as the representative result. To estimate the fatigue life (N), the maximum strains (resulting from the applied load of 750 N) were applied in a sinusoidal loading pattern and correlated with established strain-life (ε–N) curves, which were derived according to the Uniform Material Law^22^. For the displacement at the fracture site, a total force of 750 N was applied, consisting of a reduced load of 600 N and an additional opposing tensile force of 150 N. The latter represents the load induced by lifting the affected leg during gait, which is approximated as the weight of a 15 kg leg. The results are expressed as the maximum reduction and enlargement of the fracture gap with respect to the unformed geometry. The following generally applies to Cx and Cy: the higher this value is, the stiffer the structure becomes, and the less it deforms elastically when force is applied. For Gv, the following generally applies to the same material: the lower this value is, the lower the stress on the fixation structure and the longer the service life until failure. All calculations were conducted using the Ansys 19.2 software program (Ansys Inc., Canonsburg, Pennsylvania, USA). Static analyses were performed first, on which further dynamic investigations were based. All construct variants were meshed hex-dominantly with quadratic shape functions and an element size of 0.7 mm, with all fillets meshed at 0.3 mm. The cylindrical bolts were meshed using inflation. Furthermore, a nonlinear geometry was activated. Different types of contact were shown in the respective models. All screw connections and the connection between the hip prosthesis and the bone were assumed to be composites. The coefficient of friction µ was calculated as 0.15 for metal-on-metal pairing and 0.35 for metal-on-bone pairing. For the dynamic analysis, the results were determined using threshold loading and stored strain S–N curves.

### Design variants of plate osteosynthesis

**Fig. 3:**
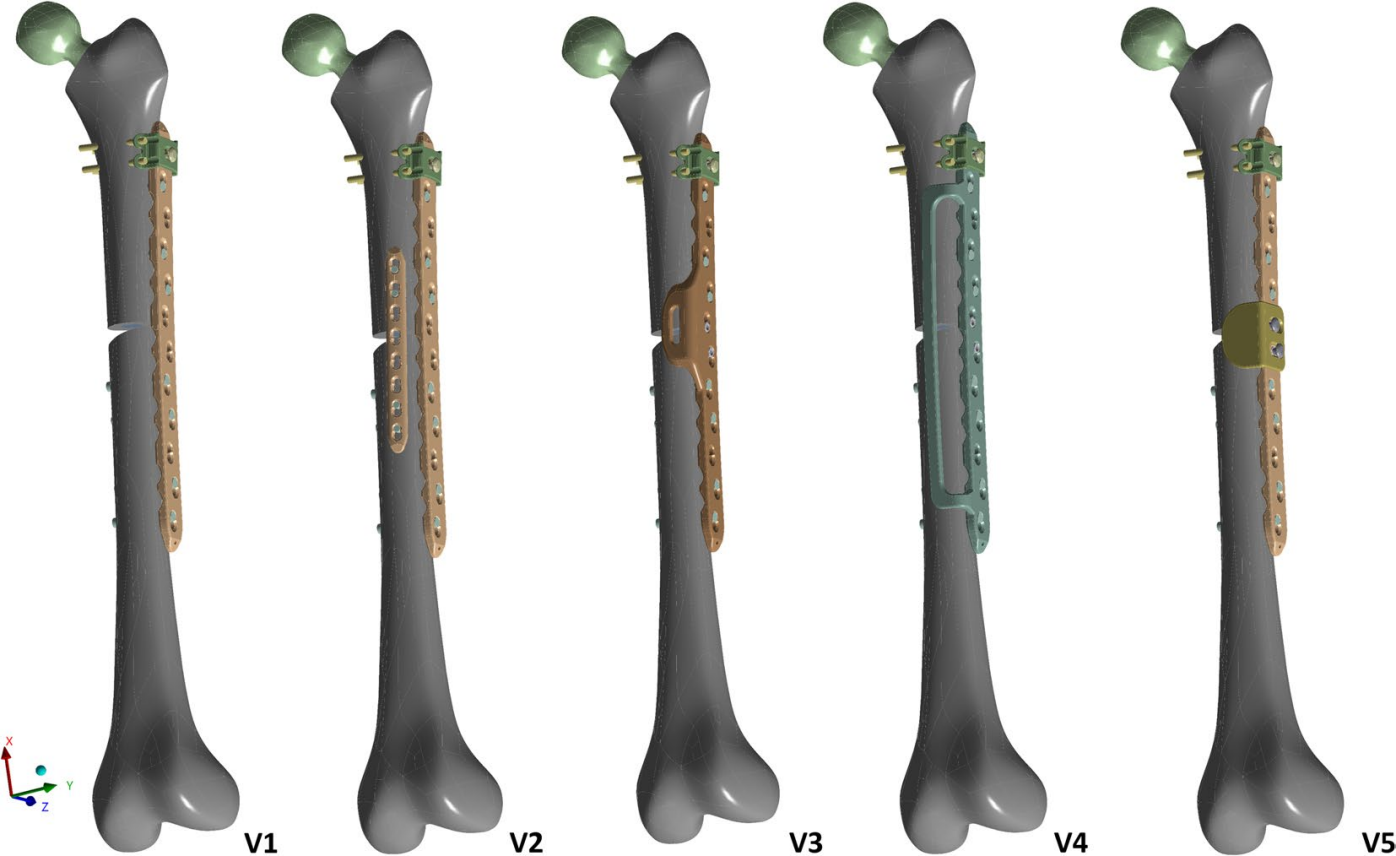
Constructing variants of a periprosthetic femoral fracture with a hip prosthesis in place with (**a**) solitary lateral LCP with the positioning of a locking attachment plate® at stem height (variant V1) and (**b**) with additive ventral LCP® (V2). The alternative plate designs include a (**c**) ventral frame at the fracture level (V3) and (**d**) over the entire length of the lateral LCP® (V4). The fifth variant is (**e**) a single fulcrum support, which is also screwed onto the existing LCP® at the fracture level (V5).

In principle, a lateral 12-hole 4.5/5.0 LCP® was used as the standard of care for each model. In variant 1 (V1), this isolated plate was fixed with bicortical screws in screw hole positions 8, 9, 11, and 12, and monocortical screws in positions 2, 4, and 5. Additionally, the plate was fixed at the level of the prosthesis stem in position 1 of the LCP® using a locking attachment plate® (LAP), which was fixed in the bone with four bicortical locking 3.5 mm screws via the LAP® tabs. Notably, to simplify all screw fixations in the FE analysis, these screws were replaced by bolt connections that correspond to the respective outer diameters of the planned screws. V2 included orthogonal double plate fixation with an additional ventral 8-hole 3.5 LCP® at the level of the fracture (fixation using four bicortical screws at plate hole positions 1, 2, 7, and 8) to absorb the ventral bending forces. Additionally, three alternative design studies were developed to optimise the LCP®, where additive ventral fixation of the plate was unnecessary. The aim was to achieve stability similar to double plating while significantly increasing load-bearing capacity compared to the lateral plate alone. Compatibility with existing plates and components is desirable. The active principle of variants 3 and 4 is the stiffening of the system using a frame or increasing the area moment of inertia in the maximum loaded cross-section of the lateral plate. This results in a new plate design based on the lateral 12-hole LCP®, which features a flanged frame in the ventral direction in the area of the fracture (V3) or over the entire length of the plate (V4), and is inserted intraoperatively from the lateral to the ventral aspect of the femur without requiring more invasive soft tissue preparation. Direct contact of the frame with the anterior aspect of the femur is unnecessary. Variant 5, on the other hand, consists of a single fulcrum support that can be screwed onto the lateral plate using the same connecting screws as those of the LAP®. The action principle here is to support the bone at the fracture level. This fulcrum support can be fixed to the lateral LCP® via a slotted hole and approximated directly, which absorbs the ventral bending moment during axial loading. The individual calculations for the implants were based on two different materials: a titanium alloy Ti6Al4V and stainless steel (medium-strength, e.g., stainless steel 1.4435) (Table 1)^23^. Studies on implant properties have shown that, in principle, titanium plates allow greater longitudinal movement and elastic deformability than stainless steel plates because of their lower modulus of elasticity. However, the yield and fracture limits, characteristic values for plastic deformation and material fractures, are greater for titanium implants. Design variants of plate osteosynthesis are reported in Fig. 3

**Table 1:** Comparison of material data regarding the yield strength, modulus of elasticity and fatigue strength of titanium and stainless steel.

Characteristic	Titanium (MPa)	Stainless steel (MPa)
Yield strength	890	500
Modulus of elasticity	110.000	210.000
Fatigue strength	500	400

## Results

**Fig. 4:**
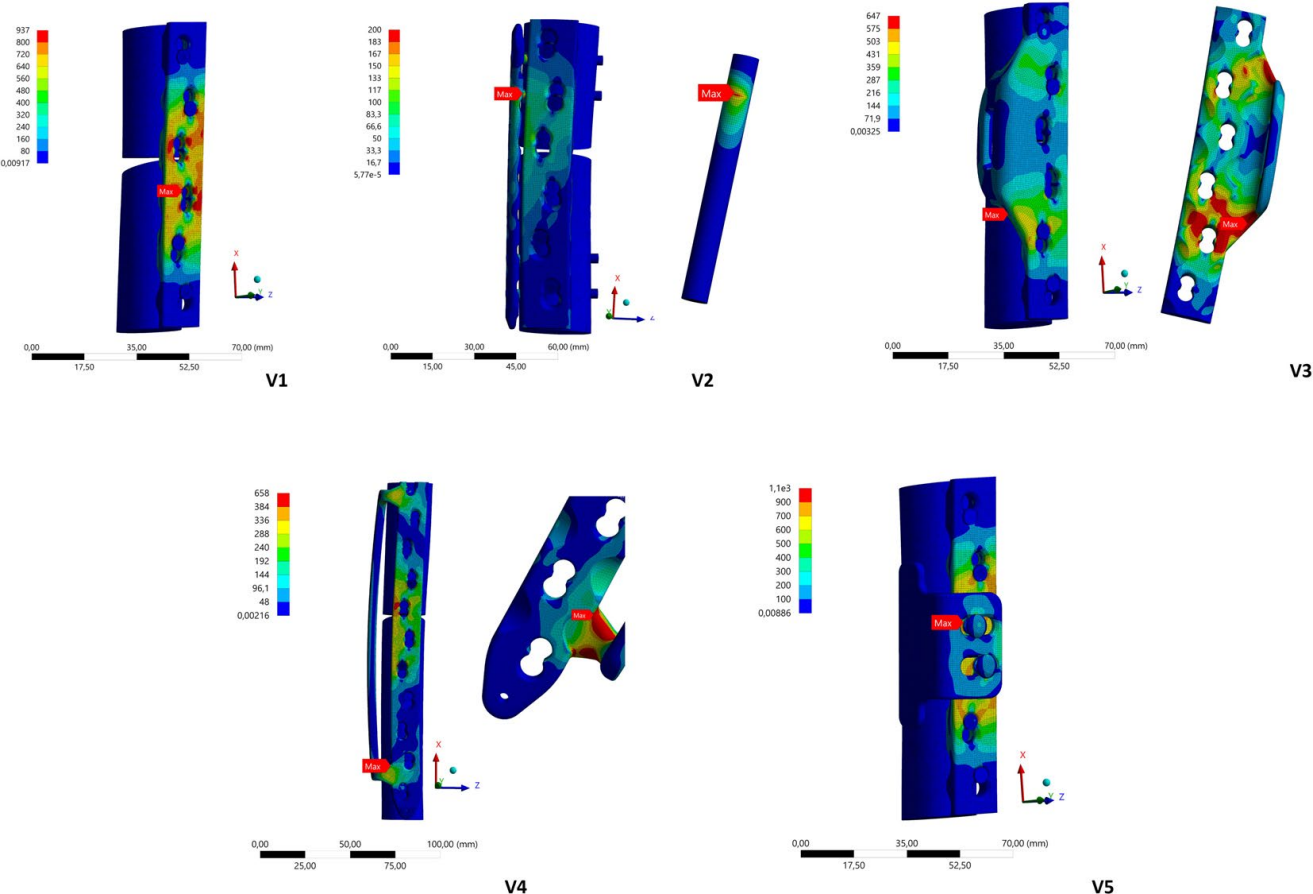
Illustration of the comparative stresses according to the von Mises (in MPa) for the solitary lateral plate (V1) when the maximum stress is reached at the plate holes close to the fracture (**a**) and for V2 in the area of the pin connection of the additional ventral plate close to the fracture (**b**). In V3 and V4, with short and long frames, the maximum comparative stress is generated on the inner sides of the outer frame connections (**c**,**d**). In V5, the maximum comparative stress is mainly observed in the bolt connection of the fulcrum support on the lateral plate (**e**).

After conducting the FE analysis, the results regarding deformation after the application of static load, equivalent stress (Fig. 4), and service life (cycles) following dynamic loading were determined.

Plastic deformation of the respective construct occurred when the yield point was exceeded. Tables 2 and 3 list the results of the static loading tests of all five constructs in the tested materials, titanium and steel. Plastic deformation was observed for V1 and V5 in the titanium variants and V1, V3, V4, and V5 in the steel plate constructs, as the yield strength was exceeded. Notably, the stiffness value is no longer valid for plastic deformation, as it applies only to the linear/elastic range. The corresponding values are shown in brackets in the tables.

**Table 2:** Results of the displacement (u), stiffness value (C, rounded) and stress G_v_ after loading the five different titanium fixation constructs.

Variation (titanium)	u_x_ (mm)	u_y_ (mm)	C_x_ (N/mm)	C_y_ (Nm)	G_v_ (MPa)
1	10,89	30,39	(69)	(25)	937
2	0,56	0,71	1339	1056	200
3	3,96	12,85	189	58	646
4	3,57	11,7	210	64	658
5	10,96	30,59	(68)	(25)	959

**Table 3:** Results of the displacement (u), the stiffness value (C, rounded) and the stress G_v_ after loading the five different steel fixation constructions.

Variation (stainless steel)	u_x_ (mm)	u_y_ (mm)	C_x_ (N/mm)	C_y_ (Nm)	G_v_ (MPa)
1	7,5	19,52	(100)	(38)	573
2	0,41	0,69	1829	1087	200
3	1,92	6,64	(390)	(113)	525
4	1,81	6,15	(414)	(122)	550
5	7,8	19,98	(96)	(38)	598

Table 4 lists the results of the load cycles and the survival time until osteosynthesis fails after dynamic loading.

**Table 4:** Results of the number of cycles after dynamic load absorption of the five different plate designs (titanium vs. stainless steel) until failure (5,000 cycles correspond to approximately one day).

Variation	Titanium (n = cycles)	Days	Stainless steel (n = cycles)	Days
1	120.000	24	40.000	8
2	1.000.000.000	fatigue- resistant	1.000.000.000	fatigue- resistant
3	5.000.000	1.000	260.000	52
4	4.200.000	840	200.000	40
5	116.000	23	37.000	7

**Fig. 5:**
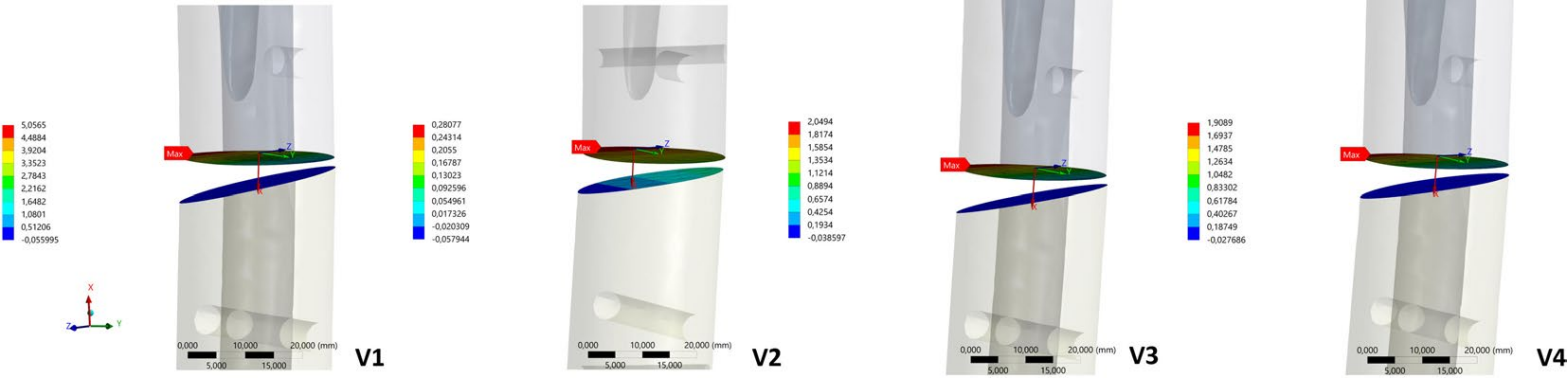
Illustration of the medial defect reduction in the area of the fracture with the respective maximum change after axial load application in mm for V1 to V4 in the titanium variants (**a**–**d**; omission of V5, as the results are identical to those of V1).

Regardless of the material variants, the orthogonal plate arrangement V2 can be regarded as fatigue-resistant. The constructs V3 and V4 exhibited significantly longer survival times than the solitary lateral plate for the steel and titanium variants. While V1 is predicted to fail after 120.000 cycles/24 days (titanium) or 40.000 cycles/8 days, the in-house developed designs V3 and V4 are only expected to fail much later, hence exceeding the duration of timely fracture healing of approximately 8 – 12 weeks, particularly in the titanium variant. Conversely, V5 has nearly identical prognostic failure times to V1. Similar to the findings regarding plate construct deformation, the isolated lateral plate osteosynthesis (V1) and the V5 variant with additional fulcrum support at the fracture level demonstrate the greatest positive strain in the fracture area compared to all other constructs, in addition to the negative strain in terms of compression following static load application, with significant narrowing of the defect zone on the medial side (Table 5), even during simulated leg lifting. Figure 5 depicts the change in the fracture gap after load application.

**Table 5:** Results of the change in the distance in mm by which the fracture zone decreases on the maximum medial side when the load is applied and increases on the maximum medial side when the leg is lifted, as well as the percentage total elongation in the fracture gap for the five different constructs in the titanium and steel variants.

Variation	Gap reduction (mm)		Gap enlargement (mm)		Total elongation (%)	
	Titanium	Stainless steel	Titanium	Stainless steel	Titanium	Stainless steel
1	4,9	2,2	0,6	0,3	55	25
2	0,3	0,3	0,1	0,0	4	3
3	1,9	1,0	0,3	0,2	22	12
4	1,8	0,9	0,3	0,2	21	11
5	4,5	2,3	0,5	0,3	50	26

## Discussion

As the number of periprosthetic femoral fractures continues to rise, the need for biomechanically optimised osteosynthetic stabilisation procedures to ensure safe bone healing while retaining the inserted prosthesis is becoming increasingly urgent. The stability of the constructs is highly dependent on the specific prosthesis or plate configuration. The aim is to create a load-bearing construct with the best biomechanical expansion behaviour for callus fracture consolidation. The present FE model study, confirms the previous assumption of the unfavourable biomechanical properties of isolated lateral plate osteosynthesis for the fixation of periprosthetic femoral fracture-type UCS IV.3-C concerning deformation and durability^10^. This plate variant (V1) exhibits rapid failure, characterised by plastic deformation of the plate, even under static force application, and represents the weakest construct in this FE analysis. These results are also consistent with the outcomes of earlier clinical studies reporting osteosynthesis failure with the isolated application of a lateral plate^14^.

Compared with purely lateral plate osteosynthesis (V1), the newly developed plate constructs featuring an additional anterior frame in the fracture zone (V3) or extending over the entire plate length (V4) demonstrate comparable results and greater stiffness, alongside a significantly increased service life that, assuming 5,000 gait cycles per day, far exceeds the regular callous fracture healing time frame of 10–12 weeks. Furthermore, in a clinical context, extensive soft tissue dissection of the anterior femur is not required to secure these plate constructs. The most stable and stiffest construct in this FE analysis, with the highest failure load, is the variant with an orthogonal, twofold plate arrangement (V2), which is consistent with the results of other studies^24,25^. To implant both plates intraoperatively requires a more invasive preparation of the quadriceps femoris muscle ventrally, with corresponding soft tissue compromise and impairment of periosteal perfusion.

In contrast, the function of isolated fulcrum support above the fracture (V5), with a direct stop on the bone to absorb the deformation of the loaded femur anteriorly, reveals almost identical results to purely lateral stabilisation. This principle has, therefore, yet to be proven as an improvement, despite sharing a similar basic biomechanical principle with variant 3. In V3, however, the anterior frame is directly connected to the lateral LCP. As a result, this reinforced section acts like angle iron, significantly strengthening the construct. In contrast, in V5, the local anterior fulcrum support is fixed via one-point fixation with a screw in a combination hole, which is fundamentally a weak biomechanical point. The plate exhibits the same near-time failure pattern as V1, regardless of the material used. Notably, compared with steel, the failure load of titanium implants increased further across all the variants. However, because steel has a higher modulus of elasticity, the steel variants exhibited lower deformation than the tests conducted with titanium alloys.

This investigation also compares biomechanical studies concerning the stability and failure load of plate osteosyntheses in patients with periprosthetic fractures. For example, the working group of Takahashi et al. analysed plate configurations of UCS IV.3-B1 fractures via finite element analysis and concluded that the orthogonal plate arrangement offers the highest stability, with a significant reduction in the stress concentration of the lateral plate at the fracture level; however, these data focus solely on direct, postoperative primary stability^25^.

In the experiments by Lenz et al. on cadaver femora, double plating (lateral/ventral) with two additional LAPs in the area of the prosthesis stem was tested^19^. The load was first applied statically and then dynamically and cyclically increased. In the work of Wähnert et al.^20^, an artificial bone with a long lateral LCP, a shorter ventral LCP, and an LAP was used, similar to the approach used in the present investigation. The load was first applied statically and then dynamically with a cyclical increase. The results of these two studies regarding the location of the maximum material stress are comparable to the results of this study. When an additional ventral plate (V2) was used, the maximum comparative stress was found in the area of the screw connection close to the fracture. In this study, such increased values were also found in construct variants V3 and V4 on the respective inner sides of the outer frame connections.

There are also other biomechanical approaches in the literature for making osteosyntheses on the femur more stable. This is particularly true when no interfragmentary contact can be established due to fracture morphology or a bone defect in the fracture area. A study by Lenz et al. from 2021 emphasised the biomechanical advantage of a second anteromedially helical plate, which is fixed with screws at both the proximal and distal ends of the femur. The analyses revealed a significant increase in axial stiffness and torsional stiffness, particularly regarding the failure load of the lateral plate, even if the minimally invasive implantation of this anteromedial plate construct must at least be called into question and causes identical problems of plate fixation around the prosthesis stem with the prosthesis in place, particularly on the proximal femur.

In addition to the issues of stability and failure load of various plate constructs, optimal elongation behaviour in the fracture gap plays an essential role in callous fracture consolidation, which is intended for multi-fragmentary fracture cases or periprosthetic femoral fracture-type UCS IV.3-C with a medial defect. This study also analyses the construct that allows the necessary elongation in the fracture gap to generate interfragmentary relative movement, according to Perren’s elongation theory^17^. Perren et al. suggest that adequate vibration behaviour is ideally achieved by elongating the fracture gap between 5 and 30%^26^. However, regarding secondary fracture healing in this test model, the orthogonally arranged double plate variant 2 is too stiff in the titanium and steel variants (4% vs. 3%), resulting in insufficient interfragmentary relative movement. Fracture consolidation can only happen with a delay or failure to appear together in pseudarthrosis formation.

Plate variants 3 and 4, on the other hand, demonstrate optimal elongation behaviour in the fracture gap for both material variants, facilitating callous healing after load absorption and under tensile loading, with total percentage elongations of 22% and 21% (titanium) and 12% and 11% (stainless steel), respectively. These newly developed constructs, therefore, represent stable fixation until regular fracture healing, providing an adequate service life, as they are associated with a reasonable degree of bridging stability, satisfactory elongation behaviour according to Perren in the fracture area, and less ventral soft tissue dissection on the femur to promote bone consolidation.

Several limitations exist within this study. The FE model was conducted with an axial load of 750 N and a tensile load of 150 N, without simulating a natural gait cycle or pathological load peaks that could occur during a fall event. Consequently, the results may diverge from in vivo measurements due to simplifications, material differences, and the idealisation of material stability. Despite this, it remains possible to categorise the duration until material failure, while acknowledging the stated limitations. Moreover, the conversion of screw connections to bolt connections in the newly developed plate constructs means these connections cannot be mechanically assessed. Hence, it is not feasible to evaluate the failure of a thread or the tearing of screws from the bone or plates. Other fixation techniques, such as combining cerclage and screws, were also not considered in this study. Furthermore, a limiting factor is the use of an artificial bone model to simulate in vivo conditions, as its properties significantly differ from those of a human femur. Ultimately, sufficient callus formation due to the enhanced resilience of the novel plate constructs can only be inferred, as various factors influencing the phases of secondary fracture healing were not taken into account in this study.

In the future, the design of the attachment devices will be optimised in terms of shape and geometry during further development via the FEM. The construct will be manufactured and tested in additional biomechanical in vitro experiments on cadaver femora to assess its clinical and practical effects.

## Data Availability

the datasets generated during and/or analysed during the current study are available throughout the manuscript.
